# Efficient neuroanalytic local–global learning with dual-branch CNN–lightweight Swin Transformer for Alzheimer’s disease analysis

**DOI:** 10.3389/fncom.2026.1972923

**Published:** 2026-10-29

**Authors:** Saddam Hussain Khan, Rashid Iqbal, Saeed Alqahtani, Rafi us Shan, Abdulaziz Alhefdhi, Bader Alkhamees, Karim Gasmi, Arshad Ali

**Affiliations:** 1 Interdisciplinary Research Center for Smart Mobility and Logistics, King Fahd University of Petroleum and Minerals (KFUPM), Dhahran, Saudi Arabia; 2 Department of Computer Systems Engineering, Artificial Intelligence Lab, University of Computing Sciences & Information Technology (UCSIT), Swat, Pakistan; 3 Department of Radiological Sciences, College of Applied Medical Sciences, Najran University, Najran, Saudi Arabia; 4 Faculty of Computer Information Science, Higher Colleges of Technology, Abu Dhabi, United Arab Emirates; 5 Department of Computer Engineering and Information, College of Engineering - Wadi Addawasir, Prince Sattam bin Abdulaziz University, Al-Kharj, Saudi Arabia; 6 Department of Information Systems, College of Computer and Information Sciences, King Saud University, Riyadh, Saudi Arabia; 7 Department of Computer Science, College of Computer and Information Sciences, Jouf University, Sakaka, Saudi Arabia; 8 Faculty of Computer and Information Systems, Islamic University of Madinah, Madinah, Saudi Arabia

**Keywords:** Alzheimer’s disease, computer-aided diagnosis, convolutional neural network, lightweight deep learning, NeuroCST, spatial attention, structural MRI, SwinLite Transformer

## Abstract

Alzheimer’s disease (AD) is one of the main causes of dementia, where early detection is critical for postponing the progression of the disease and timely treatment. Recent computer-aided diagnosis using deep learning with structural MRI has shown optimal performance; however, the rising number of model parameters and high computational requirements make the deployment challenging in resource-limited environments. This study proposes an efficient deep learning framework named “NeuroCST” based on a dual-branch network architecture incorporating an enhanced EfficientNet-based CNN and a Lightweight Swin Transformer working independently to capture local and global information of AD. The enhanced Efficient Convolution branch extracts fine-grained morphological features using depthwise separable convolution and residual learning for texture capturing. Consequently, the customized SwinLite Transformer branch models global contextual dependencies using Efficient SwinLite Transformer blocks with attention, shifted-window partitioning, and MLP. These dual-branch diverse feature vectors are then concatenated and processed by a spatial attention mechanism, NeuroCST-SA, for optimal pixel selection to reduce redundancy and discriminate minor contrast and texture inter-class variation. The proposed NeuroCST is evaluated on the public Kaggle AD dataset, which contains 6,400 original and 33,984 augmented MRI images (40,384 in total) in four classes: Mild Demented, Moderate Demented, Non-Demented, and Very Mild Demented. The NeuroCST-SA performs better on diverse experimental setups, both augmented and original, than existing CNNs and ViTs, achieving 98.42% accuracy, 98.50% sensitivity, 98.60% precision, and an F1-score of 98.55% (mean, with a standard deviation of 2.49% across holdout cross-validation). The base NeuroCST model has 15.3 million parameters and 5.2 GFLOPs, and the NeuroCST-SA variant has 14.8 million parameters, 4.9 GFLOPs, and a processing time of 11.4 ms per image. The performance demonstrates the effective integration of CNN and transformer in capturing diverse local–global features for robust AD classification.

## 1 Introduction

Alzheimer’s disease (AD) is a leading cause of dementia, which is a neurological disease resulting in the deterioration of cognitive functions (Roberson and Mucke, 2006). One of the conditions that precedes AD and carries an increased risk of its development is mild cognitive impairment (MCI) (Longhe, 2020). The pathogenesis of AD is based on the accumulation of tau and amyloid-beta proteins that lead to the destruction of brain cells. The two most typical forms of protein accumulation in the brain are amyloid-beta senile plaques and tau neurofibrillary tangles. As AD develops, patients begin to lose their self-reliance, making their lives impossible without any assistance in performing basic activities of daily living. That is why AD has become one of the most crucial global health issues. Six million people have AD at present, and by 2050, the number of patients will increase twofold to thirteen million people (Goenka and Tiwari, 2022). These figures bring about the increased cost of treating AD, which is estimated at $1.1 trillion as per the calculations of the Alzheimer’s Association (Atri, 2019). Therefore, CAD systems that use advanced algorithms along with different types of medical data can help detect and diagnose the disease (Khan and Pachori, 2023). Such systems play an essential role in improving diagnostic efficiency, reducing clinician workload, and accelerating the diagnostic process (Khan et al., 2022). Given the continually rising prevalence of AD, the demand for effective CAD systems capable of meeting corresponding diagnostic requirements has grown correspondingly (Khan et al., 2025).

Several imaging modalities are employed to monitor medical challenges, including diffusion tensor imaging (DTI), structural MRI, PET, and functional MRI (Vemuri et al., 2012). Numerous sMRI-based algorithms have been developed for CAD systems in parallel with advances in machine learning. The process of using machine learning for sMRI analysis typically includes segmentation into ROIs as per a known template structure (Khan et al., 2023). Features such as changes or structural differences within the ROIs that are deemed significant to the progression of AD are extracted. The identified features are then fed into a machine learning classifier such as a support vector machine or random forest to develop the diagnostic model (Hosseini-Asl et al., 2016). Such a process leads to the identification of unique patterns and interactions within the brain’s structures, which are used as a predictive framework for diagnosing AD (Gao et al., 2017).

One of the classes of deep learning (DL) architectures that shows great promise in medical image analysis is convolutional neural networks (CNNs) (Mehmood et al., 2026). Unlike traditional approaches that use handcrafted feature extraction, today CNN models are increasingly used in image analysis and disease detection due to their ability to learn and extract relevant features directly from the input; in addition to that, the availability of sMRI data enhances this ability. CNN models belong to a subset of deep neural networks, making them well-suited to detect subtle distinctions between images of the same category, something that requires human expertise otherwise. Transformer-based models like the Vision Transformer (ViT) (Hameed et al., 2024; Zahoor et al., 2026) have also become increasingly popular in CAD research, including AD detection (Khojaste-Sarakhsi et al., 2022). Unlike learning a global representation of long sequences, these architectures make use of the self-attention model to identify dependencies between input elements. In order to overcome the problem of the loss of important data that is associated with manual feature selection, DL methods are preferred over traditional feature extraction techniques for the processing of sMRI images (Wen et al., 2020). However, the DL framework often fails when working with small datasets where the number of extracted features is equal to or larger than the dataset itself. In this case, DL models will quickly overfit during the training process and hence perform poorly on unseen data. Current research therefore tends to focus on training entire 3D architectures that can learn how to extract important information automatically to boost the accuracy of AD classification. Other methods involve the use of both the classifier and another module for extracting high-variance regions. However, using and managing these complex architectures on high-performance computers poses many challenges.

Even though there have been promising results from DL approaches, there have been some challenges encountered while evaluating the performance of the frameworks, mainly due to the lack of multi-center test datasets used in previous studies. Moreover, comparing different approaches is difficult due to the lack of uniformity in screening criteria for the data, leading to varying test sets between the studies. CNNs and Transformers provide complementary representations for AD classification. CNNs offer strong local inductive bias and translation equivariance, enabling fine-grained morphological and textural feature extraction, whereas self-attention captures long-range dependencies and broader spatial context (Chen et al., 2026). Accordingly, CNN–Transformer hybrids employ parallel dual-branch, late-fusion to integrate local and global representations. Moreover, parallel architectures preserve branch-specific representations before fusion, while attention-based fusion further emphasizes informative features and suppresses redundancy. Motivated by this complementarity, NeuroCST adopts parallel enhanced EfficientNet-CNN and Lightweight SwinT branches to independently learn local morphology and global context, followed by feature concatenation and NeuroCST-SA for post-fusion spatial refinement. Using an early-stage CNN and two-stream network architecture, 3D inputs are first converted to 2D feature maps using the subject-level method. This encoding allows the framework to keep the volumetric data and therefore makes it easier for SwinT to extract the features. For the purpose of saving computation and parameters, efficient designs are introduced in the SwinT and convolutional modules to prevent overfitting that can arise due to a lack of sufficient data. Contributions of the study are given as:

- An efficient hybrid framework, NeuroCST, is proposed, integrating an enhanced EfficientNet-based CNN with a Lightweight Swin Transformer ‘SwinLite’ in a parallel dual-branch configuration to achieve competitive diagnostic performance. The proposed NeuroCST-SA comprises: (i) lightweight customization of the Swin Transformer ‘SwinLite’, (ii) a target-specific enhanced EfficientNet convolutional pathway, (iii) feature map squeezing and excitation of each CNN-transformer for local–global representation learning, and (iv) A new spatial attention module.
- The enhanced Efficient Convolution branch extracts fine-grained morphological details to capture local features, while the customized SwinLite Transformer learns global contextual information. The EfficientNet-based CNN branch and the Lightweight Swin Transformer branch work in parallel to extract, respectively, fine-grained local morphology and long-distance global context, improving the ability of the framework to represent abstract high-level features.
- A feature map concatenation approach is adopted to combine the representations obtained by the CNN and transformer branches to achieve an enhanced feature space. Moreover, a new post-fusion spatial attention module, NeuroCST-SA, is introduced to facilitate optimal pixel selection, helping to discriminate the subtle inter-class contrast variations shared among different ADs.
- Finally, the generalization of the proposed NeuroCST-SA has been evaluated on the publicly available Kaggle dataset with 40,384 augmented and original 6,400 MRI images in four diagnostic classes: Mild Demented (MildD), Moderate Demented (MD), Non-Demented (ND), and Very Mild Demented (VMildD), and outperforms current CNNs and ViTs.

The rest of this manuscript is organized as follows. Section 2 provides a literature review, and Section 3 describes the proposed NeuroCST framework. Section 4 presents the dataset experiment setups. Section 5 presents and discusses results, including ablation studies. Finally, Section 6 concludes the manuscript and outlines directions for future research.

## 2 Related studies

This section provides a concise review of prior automated AD diagnostic approaches based on sMRI and DL, followed by a discussion of lightweight convolutional and attention-based techniques relevant to the proposed framework (Chen et al., 2025). Prior DL-based diagnostic approaches have been categorized into four groups: 2D subject, patch, ROI, and slice-level (Wen et al., 2020). 3D patch-level approaches instead employ identification algorithms operating on whole-brain sMRI, or leverage prior anatomical knowledge to determine the ROI most relevant to the diagnostic objective. Alternative approaches train self-supervised architectures, such as autoencoders or GANs, on specific medical imaging datasets (Mendoza-Léon et al., 2020). Previous slice- and subject-level methods pretrain on natural data such as ImageNet before fine-tuning on domain-specific medical data, with the expectation that cross-domain knowledge transfer may mitigate overfitting. Kang et al. (2021), for instance, employed a deep convolutional GAN (DCGAN) to train on coronal-plane 2D slices in an unsupervised manner, yielding an area under the curve (AUC) of 0.897 and an accuracy of 90.36%.

Zhu et al. (2021) achieved 92.4% accuracy on the AD Neuroimaging Initiative (ADNI) dataset by partitioning whole sMRI volumes into non-intersecting 3D image segments, comparing AD and NC groups, and selecting segments exhibiting higher t-values as training data for AD/NC classification. Lian et al. (2022) trained a fully convolutional network (FCN) on thirty-six high-variance 3D image segments to generate disease-risk maps corresponding to AD-associated regions, achieving accuracies of 91.9 and 89.8% on the Australian Imaging Biomarkers. Hedayati et al. (2021) used an unsupervised convolutional autoencoder to extract features and then classified these features with another CNN, obtaining 95% accuracy on ADNI data.

### 2.1 Efficient convolution and attention mechanisms

CNNs get their latent representations via multiple convolutions stacked on top of each other. While increasing the number of convolution layers for feature extraction is one efficient way to increase performance, this increases computational cost and parameters. Therefore, several lightweight mechanisms have been designed to increase training and inference efficiency without decreasing performance. The group convolution mechanism in AlexNet (Krizhevsky et al., 2012), for instance, decreases computational cost through partitioning convolution kernels and feature maps into several groups. Depth-wise separable convolution proposed by Howard et al. (2019), on the other hand, reduces both computational cost and the number of parameters by using depth-wise and point-wise convolutions. Similarly, DenseNet and GhostNet (Han et al., 2020; Huang et al., 2016) mechanisms take advantage of the idea that forward propagation in DL architectures results in many redundant feature maps. By minimizing the feature maps obtained via convolution and applying linear projection to recover redundant features, DenseNet and GhostNet reduce computational cost without any loss in efficiency. Furthermore, DenseNet utilizes feature reuse by merging feature maps obtained by the first layers with those obtained by deeper layers. Inspired by this trend, Dosovitskiy et al. used ViT, adapting the transformer mechanism originally proposed for NLP tasks to computer vision through splitting images into tokens.

Unlike conventional CNN architectures, ViT frameworks employ self-attention mechanisms to encode interactions among distinct image segments, thereby extracting image information without reliance on convolution operations (Xu et al., 2015). In ViT, the conventional self-attention mechanism identifies task-relevant regions by assigning weights to feature maps generated through linear projection; matrix multiplication is then performed between these projected feature maps to yield attention maps. Owing to the matrix multiplication operations involved, the computational overhead scales as O((hw)^2^), where h and w denote the image’s height and width dimensions, respectively. To reduce this computational cost, several approaches replace global self-attention with local attention strategies or computationally lighter attention mechanisms (Liu et al., 2021; Wang et al., 2020). The SwinT (Liu et al., 2021), for instance, employs a hierarchical architecture to progressively aggregate information and reduce feature map dimensionality. Separable self-attention (Mehta and Rastegari, 2022) similarly reduces computational overhead by reformulating the matrix-based attention operation as a vector-wise element-wise multiplication, thereby lowering computational complexity to scale linearly with image size (h × w). Comparative performance evaluations with state-of-the-art methods are presented in Tables 1, 2.

**Table 1:** Performance comparison with the currently implemented method employed on Kaggle AD’s dataset.

References	Method	Accuracy	Sensitivity	Precision	F1-score
Mahmud et al. (2024)	Ensembles	92	88	90	87
Mahmud et al. (2024)	Dense CNN Ensembles	95	90	91	89
Balasundaram et al. (2023)	DL models	94.10	94.75	96.50	95.50
El-Latif et al. (2023)	Lightweight +CNN	95.93	95.88	95.93	95.90
Mahmud et al. (2024)	Custom Ensemble	96	93	89	91
Mu et al. (2023)	CNN + ViT	97.43	--	---	---
Tuncer et al. (2025)	FiboNeXt	95.93%	---	---	---
Hu et al. (2026)	CoAtNet	97.58	97.58	97.61	97.58

Values are taken from the cited studies, which used different data splits and protocols, so the comparison is indicative only.

**Table 2:** Comparison of the current method employed on OASIS and ADNI datasets.

References	Method	Dataset	Acc%	Sens%
Kong et al. (2022)	3DCN	ADNI	93.21	
El-Sappagh et al. (2022)	LSTM	ADNI	93.87	
Yu et al. (2022)	CNN	ADNI	95.40	---
Hassan et al. (2024)	RBMSDL	ADNI	96.45	96.50
Yan et al. (2025)	Hybrid-RviT	ADNI	95%	
Bordin et al. (2022)	EfficientNetB0	OASIS	80.00	---
Hassan et al. (2024)	RBMSDL	OASIS	91.99	92.00
Mahmud et al. (2024)	Ensemble (EfficientNetB3&CNN)	OASIS	96.00	---
Chakra Bortty et al. (2025)	EnsembleViT-B16 + CNN	OASIS	97.31%	

Values are taken from the cited studies, which used different data splits and protocols, so the comparison is indicative only.

### 2.2 Research gap and motivation

Although substantial progress has been achieved in AD classification through DL-based approaches, several critical limitations persist in the existing literature. First, the majority of prior studies have employed computationally expensive 3D architectures that, while capturing volumetric information, impose prohibitive training and deployment costs. Second, the reliance on large-scale pretraining on natural image datasets, such as ImageNet, does not consistently translate to improved performance on medical imaging tasks, given the substantial domain shift between natural and medical images. Thirdly, a consistent comparison among diverse techniques is quite difficult because of the lack of consistency in evaluation procedures, data screening, and the limited availability of multi-center data for testing. Fourth, while existing CNN and ViT models individually have unique advantages, they have not been utilized optimally to enable capturing both local morphological characteristics and global dependencies for diagnosing AD. Lastly, efficient computing in transformer-based architectures still poses challenges; conventional ViT models are known to be quadratic in complexity relative to input image size.

The CNN branch captures fine-grained morphological and textural patterns through a convolutional receptive field, including depth-wise separable convolution. Whereas SwinT models broader spatial dependencies using hierarchical shifted-window self-attention (Zhang et al., 2026). Their feature maps are subsequently concatenated to preserve complementary local–contextual information. Secondly, the SA mechanism incorporated into the NeuroCST-SA framework further contributes to better discrimination between different classes through selective enhancement of discriminative regions and ignoring unnecessary information. The SA mechanism refines the boosted representation by emphasizing discriminative spatial regions and suppressing less relevant or redundant responses using high weight for inter-class and balanced weight for intra-class variation. Third, the model is extensively tested on a large-scale Kaggle dataset of 40,384 MRI scans (6,400 original and 33,984 augmented) from four classes. The lightweight design of the proposed framework, with 14.8 million parameters and 4.9 GFLOPs for the NeuroCST-SA variant, renders it suitable for deployment in resource-constrained clinical environments.

## 3 Methodology

This section first describes the data sources employed in the study and provides a comprehensive account of the proposed NeuroCST (Neuro-CNN–SwinLite Transformer framework), including its overall architecture Figure 1 and the efficient design of its convolutional and SwinLite Transformer blocks.

**Figure 1:**
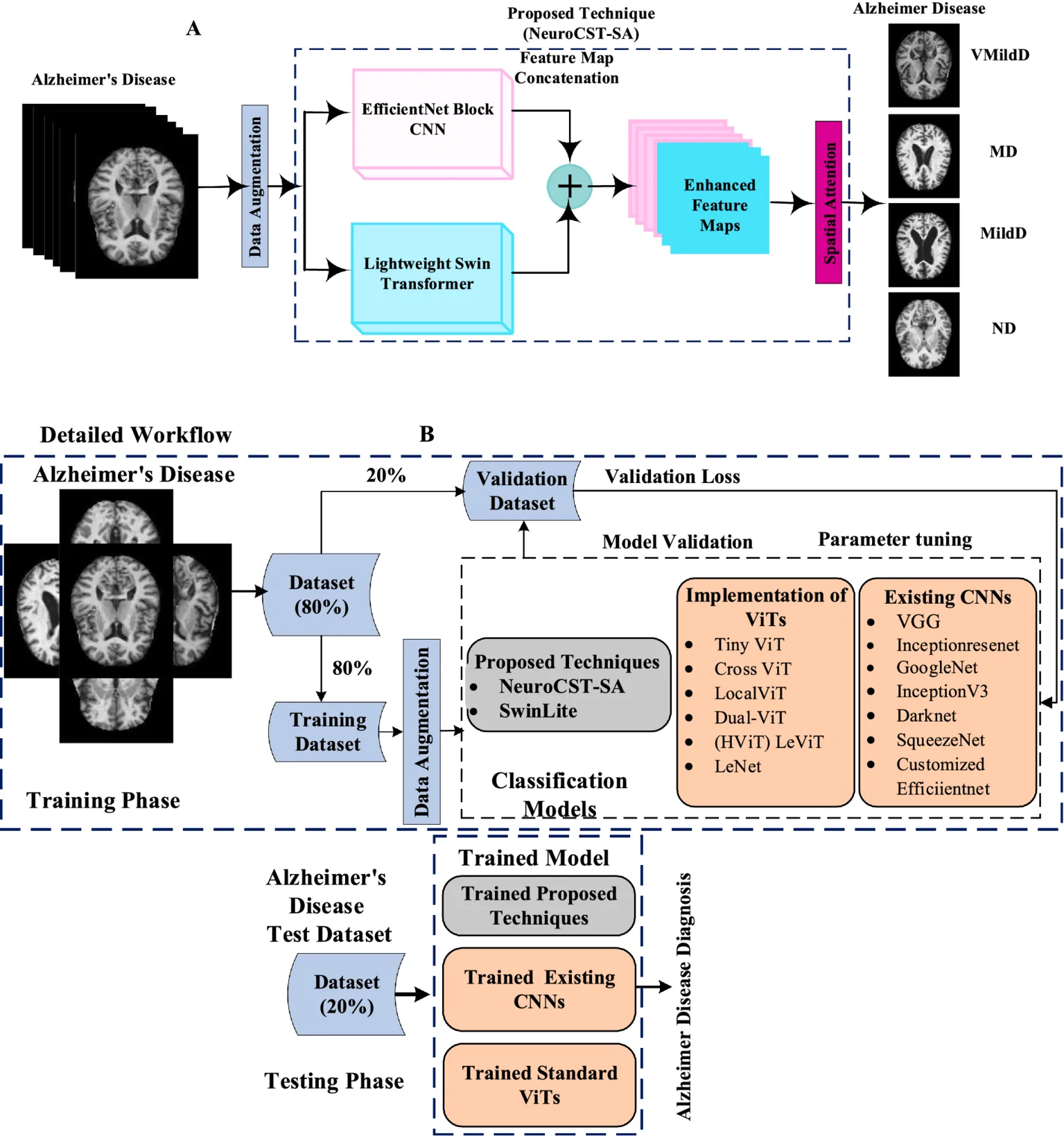
Overview of the proposed NeuroCST-SA AD classification framework.

### 3.1 Materials

AD-Multiclass Images DatasetV2 (AD-MID) (Augmented Alzheimer MRI Dataset V2, n.d.) is publicly available on Kaggle and comprises four multiclass categories: ND, VMildD, MildD, and MD, as shown in Figure 2. The dataset is derived from clinically annotated MRI cohorts originally released by the OASIS (OASIS Alzheimer’s Detection, n.d.). The OASIS dataset underwent clinical characterization that has been annotated by radiologists using Clinical Dementia Rating-based assessment and clinically diagnosed dementia status. The dataset is documented as a publicly available MRI cohort, including participants clinically diagnosed with very mild-to-moderate AD. The OASIS dataset has been categorized into four distinct classes based on the progression of AD, allowing for a nuanced evaluation of the model’s performance across various stages. Additionally, the longitudinal nature of the OASIS dataset facilitates the examination of cognitive decline patterns and predicts conversion risks over time. Each subject contributed approximately 3D NIfTI (.nii) scans, converted into 2D JPEG slices (224 × 224 × 3) using a DICOM Converter for DL compatibility. The present study performed secondary analysis only and involved no new participant recruitment or MRI acquisition.

**Figure 2:**
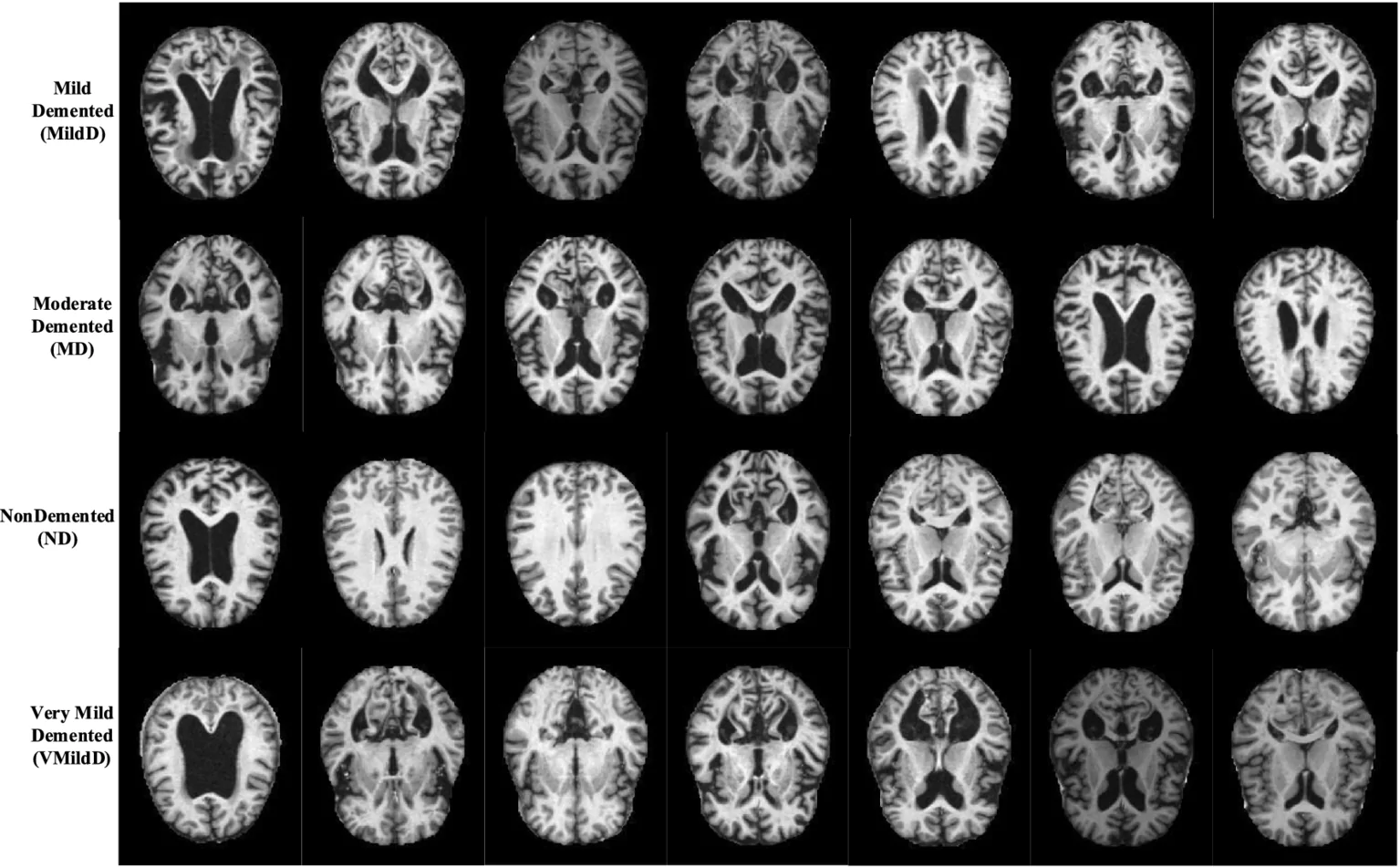
AD-labeled MRI images comprise four classes: MildD, MD, ND, and VMildD.

The Kaggle release represents an augmented and class-balanced derivative dataset comprising 40,384 MRI scans of both original (6400) and pre-generated off-the-fly augmented (33,984) scans, compared with an exclusively original dataset. Three experimental setups have been performed, categorized into two cases to assess the framework’s generalization capability and its ability to discriminate between different AD stages. In Case 1, off-the-fly augmentation, the 33,984 augmented MRIs have been employed for model training, while 6,400 original images have been kept for testing. In Case 2, with on-the-fly augmentation, only the 6,400 original images have been used and partitioned into 4,097 training, 1,023 validation, and 1,280 testing images. On-the-fly augmentation was applied exclusively to the training subset, whereas validation and testing remained unchanged from the original images. An 80:20 holdout cross-validation for training and testing in Case 2 has been adopted, where the training set is further divided into training and validation subsets for hyperparameter selection, whereas the remaining 20% was retained as an untouched test set. The selected hyperparameters using the original-image validation set in Case 2 have been subsequently cross verified on validated in Case 1 off the fly-augmented and therefore, strengthen the model training on diverse and large dataset. Moreover, the validation sets in both cases are then integrated into the training set for optimal, robust training of the models. The class distribution is summarized in Table 3.

**Table 3:** Details of the AD-Kaggle data used in the experimental setup.

Class Label	Augmented dataset		Original dataset	Total
	Training	Validation Set	Testing	
AD-multiclass images Datasetv2 (AD-MID) (Case 1)				
MildD	7,168	1792	896	9,856
MD	5,171	1,293	64	6,528
ND	7,680	1920	3,200	12,800
VMildD	7,168	1792	2,240	11,200
Total	27,187	6,797	6,400	40,384
AD original dataset (Case 2)				
MildD	574	143	179	896
MD	41	10	13	64
ND	2048	512	640	3,200
VMildD	1,434	358	448	2,240
Total	4,097	1,023	1,280	6,400

Case 1: offline-augmented images for training and validation, original images for testing. Case 2: original images only (counts before on-the-fly augmentation).

### 3.2 Proposed framework

Transformer-based models, including ViT, have been shown to achieve excellent performance in computer vision applications, such as semantic segmentation and classification tasks in recent years. However, there have been limited attempts to use transformer models for automated AD classification. In this regard, the NeuroCST framework presents a parallel dual-branch architecture that is made up of a novel improved.

EfficientNet-based CNN and Lightweight SwinT branches to allow for the extraction of fine-grained local morphological features and global contextual dependencies simultaneously. Figures 1, 3 depict how the CNN branch extracts localized morphological features that are typical of AD, such as atrophy patterns in the hippocampus, entorhinal cortex, and the regions of the temporal lobes. At the same time, the lightweight SwinT branch extracts global contextual dependencies due to its self-attention mechanism. Subsequently, the feature maps from both branches are concatenated together to provide enriched complementary features. Furthermore, in the NeuroCST-SA framework, spatial attention is introduced to weight the concatenated features spatially, so that regions that are more informative for classification are emphasized and less relevant regions are suppressed.

**Figure 3:**
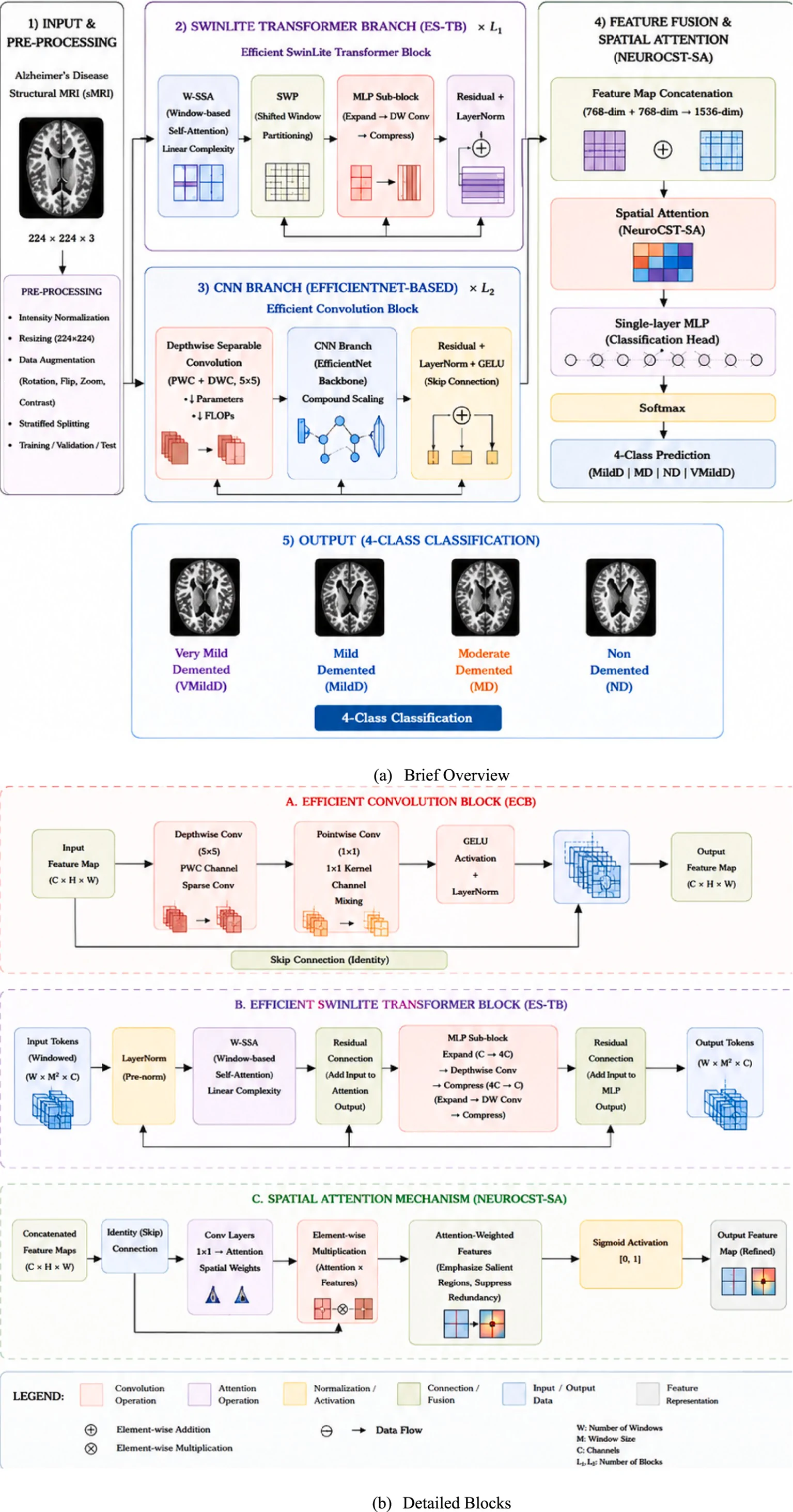
**(a,b)** Brief and detailed architecture of the proposed Efficient Convolution Block (ECB), Efficient SwinLite Transformer Block (ES-TB), and NeuroCST-SA spatial attention mechanism.

Efficient design changes in terms of reducing the computational cost and number of parameters are applied to both branches. The CNN branch applies depth-wise separable convolution (DSC) instead of the usual convolutional blocks, while the SwinLite Transformer branch applies separable self-attention (SSA) instead of the ordinary multi-head self-attention (MSA), which is linear. The architecture of the framework consists of two branches in parallel, where the CNN branch is made up of Efficient Convolution Blocks (ECB) and the SwinLite Transformer branch is made up of Efficient SwinLite Transformer Blocks (ES-TB). After processing through the two branches independently, the outputs are combined and used as inputs in a single-layer MLP to classify the inputs. Consequently, the output passes through the softmax function to produce probability outputs in the four classification classes: MildD, MD, ND, and VMildD.

### 3.3 Efficient swin transformer block

The reduction in computational overhead in the case of the SwinLite Transformer branch is due to the use of SSA instead of the traditional MSA, and this SSA works with linear computational complexity. In MSA, the query matrix (Q), key matrix (K), and value matrix (V) are computed using the linear projection layers for computing attention matrices as shown in Figure 3. In window-based multi-headed self-attention (W-MSA), where W refers to the number of windows (with the window size W = w^2^), the matrices Q, K, and V have the dimensions ${\mathbb{R}}^{W\times M2\times C}$ . By partitioning Q, K, and V into H heads, the MSA and W-MSA can compute self-attention in H subspaces; thus, the attention module can gather information from multiple representational subspaces, which improves the model’s expressiveness, as shown in Equations 1–4.

According to Raffel et al. (2020) and Hu et al. (2018), patch-relative position information is used for computing attention; this is necessary because the self-attention module cannot intrinsically consider the spatial context in feature maps. The formula for traditional W-MSA is as follows, where $Q=W_{q}X,K=W_{k}X,V=W_{v}X$ represents the weight matrices for the projection operation (Equation 1). The operation of W-MSA is defined in Equation 1. In Equation 2, $W_{0}\in {\mathbb{R}}^{C\times C}$ is the linear projection of the output after attention aggregation. For a feature map of size C × h × w, the computation cost of conventional MSA and windowed MSA. Following attention-weighted aggregation, $W_{o}\in {\mathbb{R}}^{C\times C}$ linearly projects the output. Given a feature map of size C × h × w, the computational cost of MSA and W-MSA is represented in Equation 3.


$$W_{q},W_{k},W_{v}\in {\mathbb{R}}^{C\times C}\;\;\;\;$$ (1)

$$W-\mathrm{MSA}(Q,K,V)=\text{Softmax}(QK^{⊤}+B)V\cdot W_{o}\;\;$$ (2)

$$\Omega (\mathrm{MSA})=4\mathrm{hw}C^{2}+2{\left(\mathrm{hw}\right)}^{2}C$$ (3)

$$\Omega (W-\mathrm{MSA})=4\mathrm{hw}C^{2}+2M^{2}\mathrm{hwC}$$ (4)

However, while window-located attention brings down the computational cost from O( ${\left(\mathrm{hw}\right)}^{2}$ ) to O ${\left(\mathrm{hw}\right)}^{.}$ , batch-matrix multiplication in big batches is still computationally intensive. This is why the SwinT block was redesigned to include SSA. Following linear projection with weight $W_{i}\in {\mathbb{R}}^{C}$ , SSA replaces Q with a vector input $I\in {\mathbb{R}}^{W\times M^{2}}$ , transformed into context scores $C_{s}\in {\mathbb{R}}^{W\times M^{2}}$ via softmax. Unlike W-MSA, in which attention ratios are computed for each token relative to all M^2^ tokens of K, separable attention computes context scores relative to a single latent token *L*, represented indirectly within SSA as the learnable weight $W_{i}$ . The context vector $C_{v}\in {\mathbb{R}}^{1\times C}$ is subsequently computed by weighting K with C_s_ as shown in Equation 5:


$$\begin{array}{cccc} & C_{v}=\sum_{i=1}^{M^{2}}C_{s}(i)\cdot K(i) & & \end{array}$$ (5)

With C_v_, all tokens within a given window share identical encoded contextual information, yielding a computationally less expensive analogue to the attention map produced by W-MSA. Using C_s_ and C_v_, the matrix computations characterizing standard MSA and W-MSA may be reformulated as two lower-cost element-wise product operations. This formulation extends analogously to window-based attention as represented in Equations 6, 7. Where $W_{i}\in {\mathbb{R}}^{C\times 1},W_{k},W_{v}\in {\mathbb{R}}^{C\times C}$ represent learnable weight matrices. In contrast to traditional W-MSA that calculates attention scores for all token pairs, the W-SSA algorithm substitutes the query matrix with the learned vector I, making the attention calculation an element-wise process. Window separable self-attention (W-SSA) is defined as follows (Equations 6, 7):


$$l=W_{i}X,K=W_{k}X,V=W_{v}X$$ (6)

$$W-\mathrm{SSA}(I,K,V)=\sum (\text{Softmax}(I)\cdot K)\cdot \text{ReLU}(V)W_{o}$$ (7)

$$\Omega (W-\mathrm{SSA})=\mathrm{hw}(3C^{2}+C)+2\mathrm{hwC}$$ (8)

Although W-SSA (Equation 8) $W_{o}\in {\mathbb{R}}^{C\times C}\;$ reduces computational overhead across both attention and projection components, its complexity remains O(hw). Experimental observations indicate that, despite the computational complexity of W-SSA being independent of window size (M × M), employing window-based rather than global attention yields greater convergence stability during later training stages within the present framework and objective. The proposed ES-TB, illustrated in Figure 3, comprises two sub-layers within each sub-block—layer normalization preceding W-SSA and the MLP, applied sequentially. The MLP consists of two fully-connected layers: the first expands the input token dimensionality, while the second compresses it back. Whereas the Swin-T employs r_expand = 4, this expansion factor was found to substantially increase the computational cost of the Swin-T block; accordingly, r_expand is set to 1 in ES-TB. Furthermore, because window-based attention, unlike global attention, is restricted to localized regions of the feature map and cannot capture distant dependencies, the second sub-block of ES-TB partitions W-SSA using the shifted-window partitioning strategy of Swin-T to enable connectivity across neighboring windows.

### 3.4 Efficient conv block

A conventional convolutional layer employs C_output kernels, each of dimension C_input × k × k, where k × k denotes the kernel’s receptive field (Formula 9). The dense connectivity between C_input feature maps and C_output kernels necessitates substantial multiply-accumulate operations to compute output feature maps. Given an output feature map of size h × w, the corresponding computational overhead is Equation 9:


$$\Omega (\text{standard conv})=C_{\text{output}}\cdot C_{\text{input}}\cdot h\cdot w\cdot k^{2}$$ (9)

Within the initial two CNN stages of the proposed framework, depth-wise separable convolution (DSC) is employed in place of conventional convolutional blocks, primarily to reduce the substantial computational overhead associated with dense multiply-accumulate operations. DSC comprises two components: point-wise convolution (PWC) and depth-wise convolution (DWC). The DWC operation is implemented via segmented convolution, with the number of segments matched to the channel count of the input feature maps; each DWC kernel comprises a single filter operating on a single feature map to perform a sparse convolution. PWC, by contrast, constitutes a conventional convolutional layer with a 1 × 1 kernel, enabling information exchange across feature maps while linearly projecting DWC outputs into the desired output dimensionality. The computational overhead of DSC is expressed as Equation 10. Also, the optimization of the ECB network is done by adding a residual connection as used in ResNet (Wang et al., 2017) and the pre-normalization of the SwinLite Transformer. GELU activation and layer normalization are applied in order to match the activation and normalization technique of the SwinT.


$$\Omega (\mathrm{DSC})=C_{\text{input}}\cdot h\cdot w\cdot k^{2}+C_{\text{output}}\cdot C_{\text{input}}\cdot h\cdot w$$ (10)

### 3.5 Spatial attention mechanism

An innovative spatial attention mechanism has been introduced in the NeuroCST-SA framework for efficient pixel selection and improvement in the identification of contrast patterns and inter-class differences across various AD categories. The spatial attention module works on feature maps of the dual-branch model in order to assign attention weights spatially. It helps the model to pay more attention to those regions of the image that have diagnostic significance and ignore those regions of the image that do not hold any importance for classification. It enables the framework to concentrate on those small morphological features of the brain that are indicative of AD pathology, like hippocampal, entorhinal cortex, and temporal lobe atrophy. Spatial attention weights are calculated through convolutional layers followed by a sigmoid function that results in a spatial attention map, which is then used for multiplication with the original feature maps. This mechanism leads to better results, with the NeuroCST-SA architecture having an accuracy of 98.42%, sensitivity of 98.50%, precision of 98.60%, and F1-score of 98.55% (± 2.49, standard deviation (S.D)).

### 3.6 Training and optimization

The presented NeuroCST framework has been implemented using the PyTorch DL framework. Training has been carried out using the dataset, which has been made available publicly, using AdamW as the optimizer for optimizing the parameters. The learning rate has been initialized to 1e-4, and the learning schedule has been chosen as cosine annealing decay. Cross-entropy has been used as the objective function for the multi-class classification problem. Training has been done for 100 epochs with a batch size of 32, and early stopping has been applied in order to avoid overfitting. Augmentation techniques, as discussed in Section 3.3, have been used for training the data. The NeuroCST model comprises approximately 15.3 million parameters with 5.2 GFLOPs, whereas the NeuroCST-SA variant achieves improved efficiency with 14.8 million parameters, 4.9 GFLOPs, and an inference time of 11.4 milliseconds per image.

## 4 Experiment and results

This section initially presents implementation specifics. Verification of the core network benefits over conventional 2D frameworks is subsequently performed through examination of the proposed dual-branch architecture. Numerous ablation experiments are carried out for evaluation of the techniques employed within the proposed NeuroCST (Figures 4–7). Finally, the framework’s performance is contrasted against prior studies that used the same or related public datasets.

**Figure 4:**
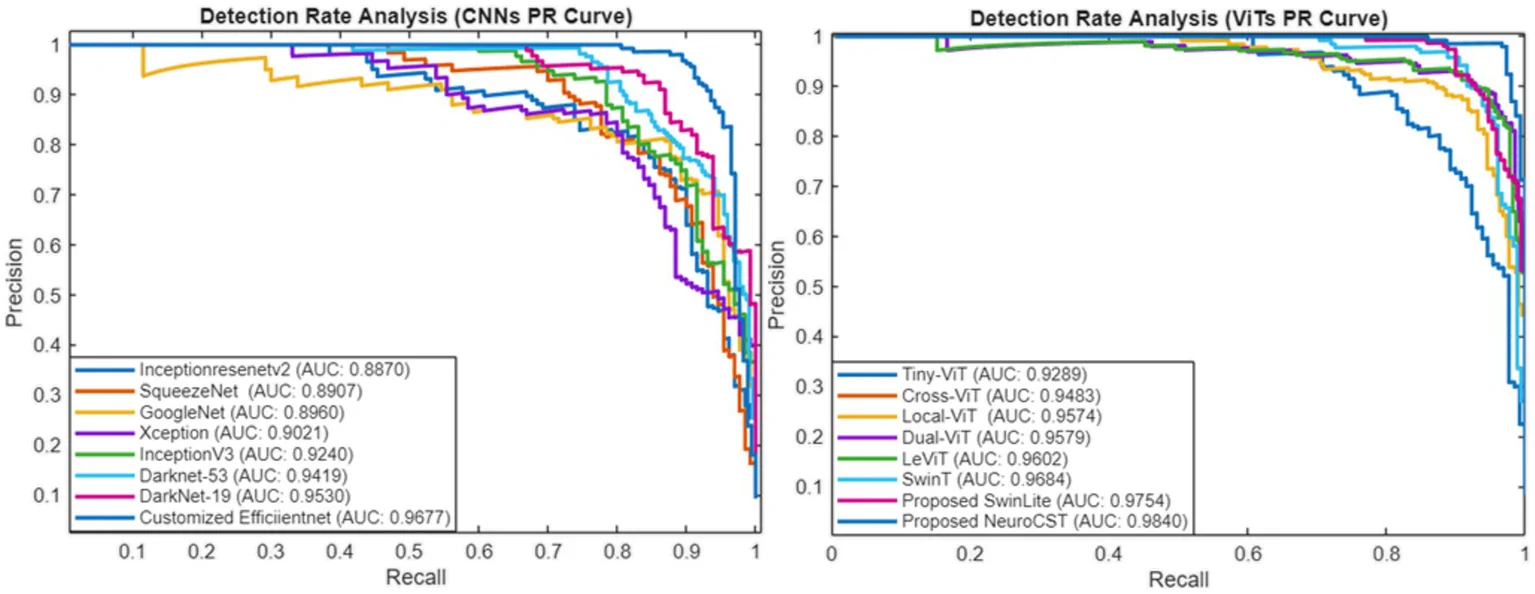
Case 1: performance on the test original set (Case 1); PR curves (demented vs. non demented) comparing the proposed NeuroCST with state-of-the-art methods on the AD-MID dataset.

**Figure 5:**
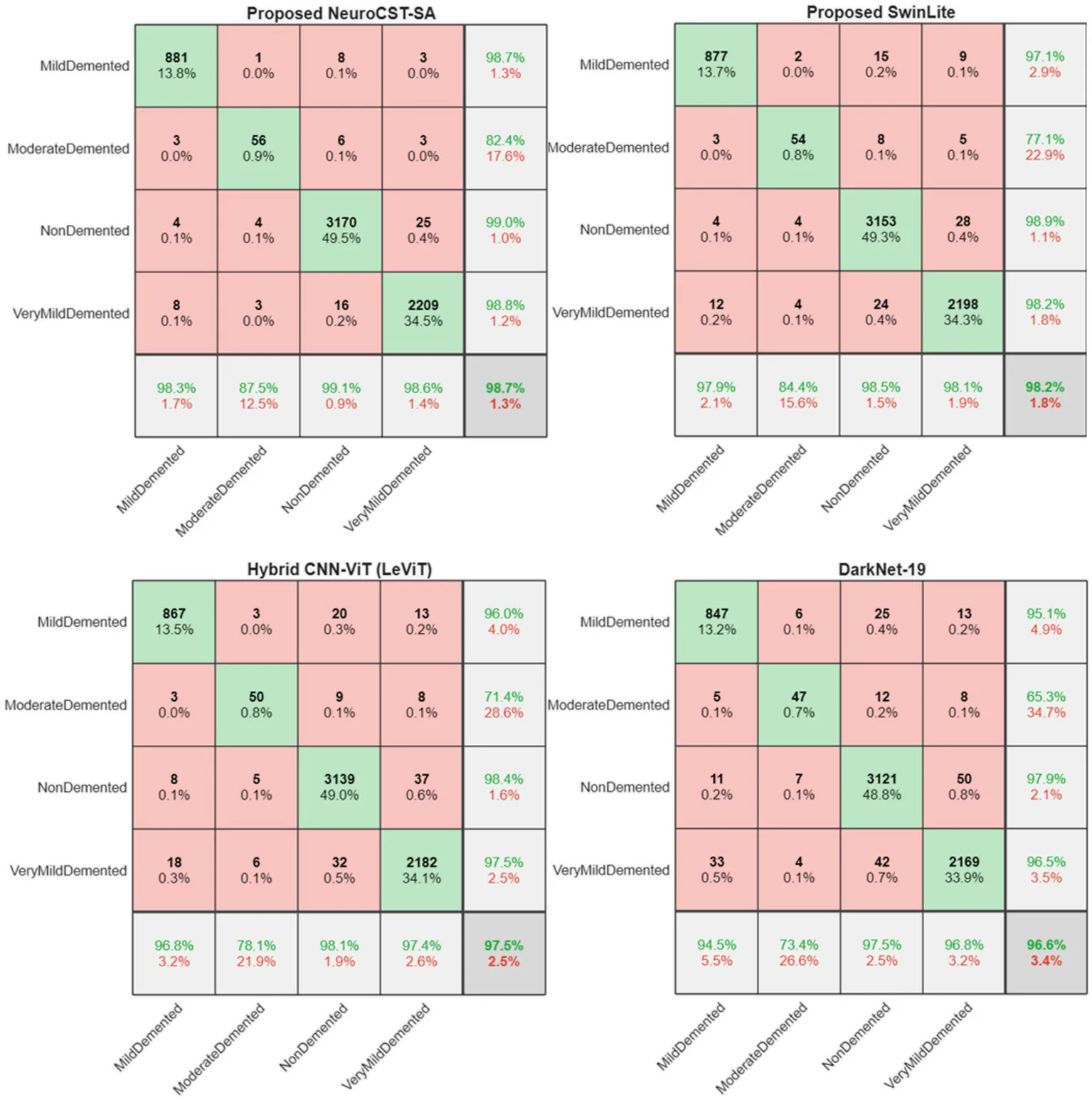
Confusion matrix for the evaluated techniques (Case 1: performance on test original set).

**Figure 6:**
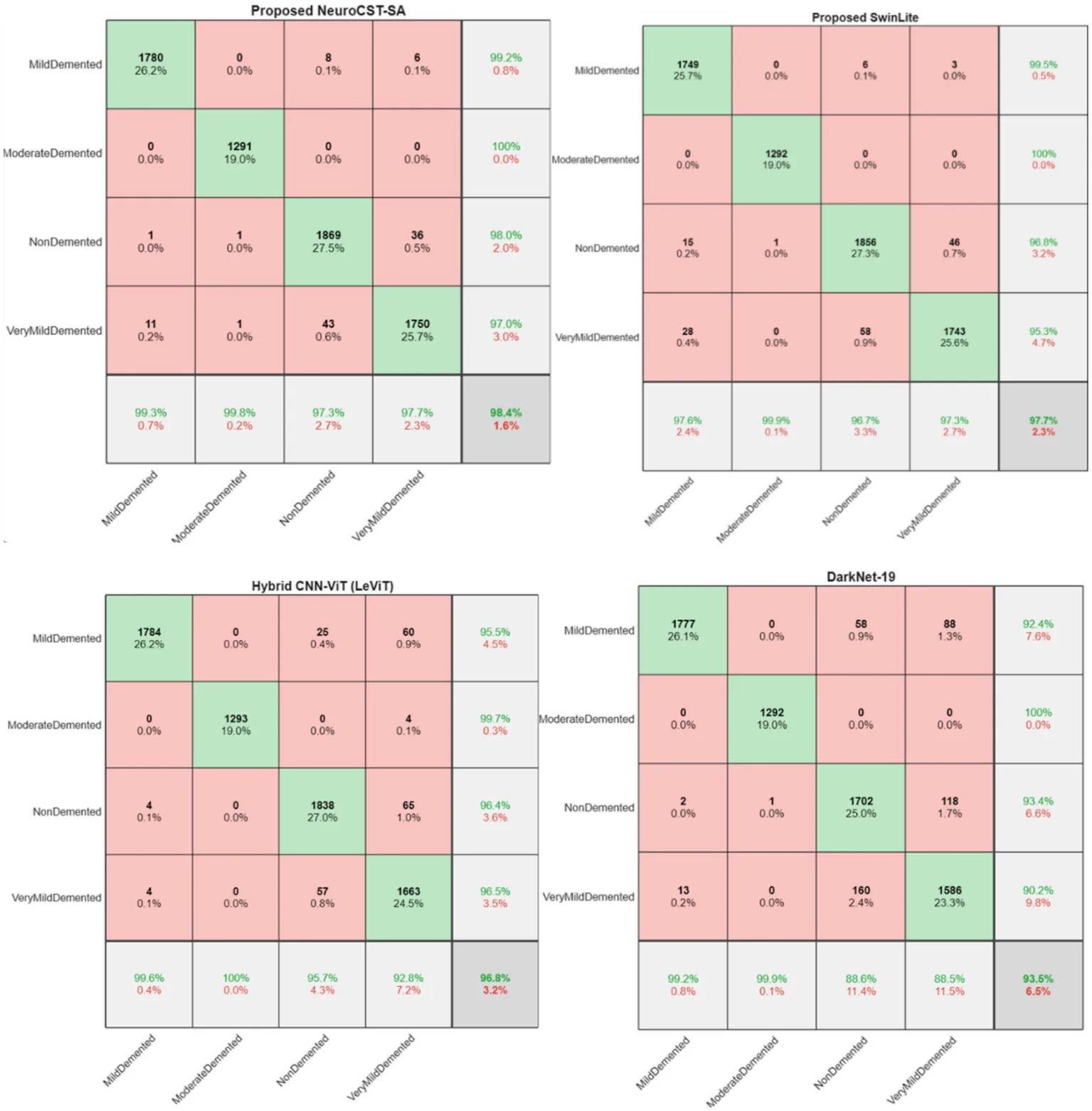
Confusion matrix for the evaluated techniques (Case 1: performance on validation set).

**Figure 7:**
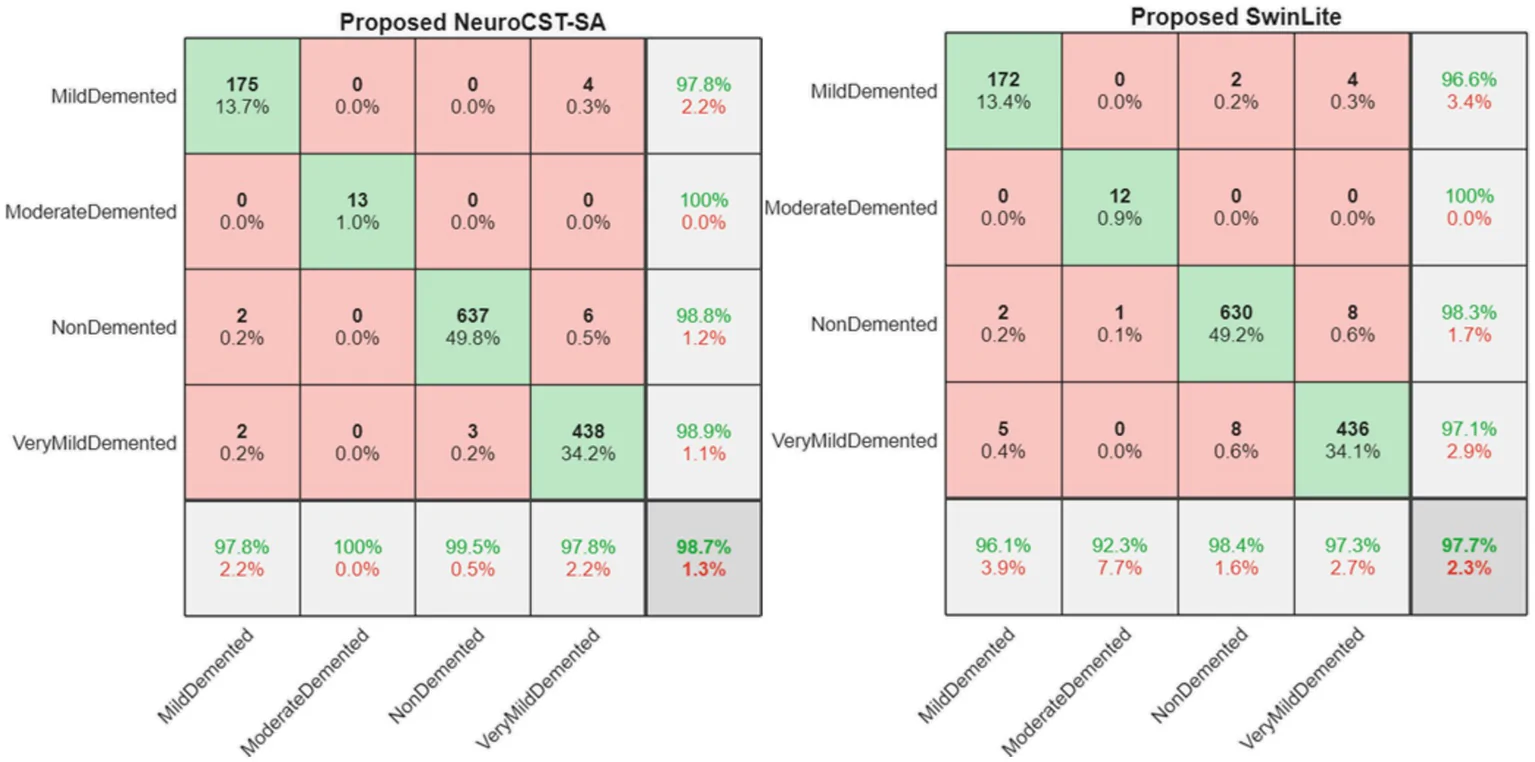
Confusion matrix for the evaluated techniques (Case 2: performance on original dataset).

### 4.1 Implementation setup

An Intel Core i5 processor with 32 GB RAM and an RTX 3070 8 GB GPU executed the training and testing tasks using PyTorch 1.10.0 and Python 3.8.8. All training data employed in the experiments originated from the AD-Multiclass Images DatasetV2 (AD-MID), as described in Section 3.1. Strengthening of the findings was achieved using holdout cross-validation for framework performance evaluation. Following the first convolution block, feature map dimensionality reached 64, subsequently doubling in size after each max-pooling layer. The patch embedding layer created 384-dimensional tokens after the previous two phases. The proposed NeuroCST underwent training using the AdamW optimizer with weight decay. Weight decay assumed a value of 0.04, and the initial learning rate was established at 1 × 10^−5^. Reduction of the class imbalance effect was achieved through measurement of the classification loss using a weighted cross-entropy function, whose weight was determined by dividing the training set’s class ratios by 2, thereby rendering the loss function more biased in favor of the underrepresented groups. Metrics including accuracy, F1-score, precision, sensitivity, precision–recall (PR) curves, and balanced accuracy served for analysis of the framework’s performance across multiple dimensions. The variability of the F1-score is reported as the standard deviation (±) across the holdout cross-validation. Equation 11 compute the computation of the Standard Error (S.E.) for the F1-score within a 95% Confidence Interval (CI), aiming to enhance the True Positive (TP) rate and minimize False Negatives (FNs) in AD’s analysis. The z-value of 1.96 represents the S.E. within the 95% CI. F1-score was chosen for this purpose because it combines precision and sensitivity and is more informative than accuracy for the imbalanced four-class data; accuracy and the AUC values are reported as complementary measures (Khan and Pachori, 2021).


$$C.I=z\sqrt{\frac{\text{error}(1-\text{error}\;)}{\;\text{Total Samples}\;}}$$ (11)

### 4.2 Ablation study

DSC and window-based separable self-attention (W-SSA) served to lighten the convolutional and SwinLite Transformer components within the proposed NeuroCST parallel dual-branch architecture. An ablation study was conducted using various component configurations, presenting the classification outcomes on the employed dataset, parameter counts, and computational costs for each configuration for assessment of the framework’s effectiveness and efficiency. The ablation results are given in Table 4 (Case 1, original test set). Replacing the standard Swin Transformer with the SwinLite version reduces the parameters from 12.4 M to 9.6 M (about 23% fewer) and the FLOPs from 4.6 G to 3.8 G (about 17% fewer), while accuracy does not decrease (97.74% for SwinLite and 97.40% for SwinT). The customized CNN branch alone reaches 97.31% accuracy with 11.2 M parameters. When the CNN and SwinLite branches are combined in parallel (NeuroCST), accuracy increases to 98.18%, which is 0.44 percentage points higher than SwinLite alone and 0.87 percentage points higher than the CNN branch alone, at the cost of 15.3 M parameters and 5.2 GFLOPs. Adding the spatial attention module (NeuroCST-SA) gives a further gain of 0.24 percentage points (98.42%) with 14.8 M parameters and 4.9 GFLOPs. These differences between neighbouring configurations (0.24 to 0.87 percentage points) are smaller than the standard deviations listed in Table 4 (± 2.49 to ± 4.14), so they should be read as trends and not as statistically established gains.

**Table 4:** Comprehensive performance comparison of proposed NeuroCST with existing state-of-the-art CNN and ViT architectures on the AD-MID dataset.

Model	Accuracy	Sensitivity	Precision	F1-Score± S.D.	Ep./Min.	Param (M)	FLOPs	Inf. (ms)
Proposed setup (Case 1: performance on test original set)								
Customized efficient CNN	97.31	97.95	95.83	96.88 ± 4.14	3.65	~11.2	~4.1	~8.2
SwinT	97.40	97.93	97.90	97.91 ± 3.12	3.70	~12.4	~4.6	~9.1
SwinLite	97.74	96.03	98	97 ± 4.02	3.55	~9.6	~3.8	~7.9
NeuroCST	98.18	97.92	98.24	98.08 ± 3.11	5.35	~15.3	~5.2	~11.8
NeuroCST-SA	98.42	98.50	98.6	98.55 ± 2.49	5.15	~14.8	~4.9	~11.4
Case 1: performance on validation set								
SwinLite	97.69	97.89	97.86	97.87 ± 3.85	3.72	~9.6	~3.8	~7.9
NeuroCST-SA	98.43	98.54	98.54	98.54 ± 3.63	5.39	~14.8	~4.9	~11.4
Case 2: AD original set								
SwinLite	97.66	98	96.04	96.98 ± 5.02	1.1	~9.6	~3.8	~7.9
NeuroCST-SA	98.67	98.85	98.77	98.81 ± 3.49	1.3	~14.8	~4.9	~11.4
Existing CNNs (Case 1: performance on test original set)								
InceptionResenetv2	86.95	79.85	84.58	82.14 ± 18.72	4.40	~55.0	~15.2	~18.9
GoogleNet	87.48	79.72	92.86	85.79 ± 15.11	5.20	~6.8	~1.5	~6.3
InceptionV3	88.59	86.2	90.34	88.22 ± 12.71	5.40	~23.9	~5.7	~10.5
DarkNet-53	88.13	87.49	90.15	88.80 ± 12.13	4.45	~41.6	~18.7	~21.8
SqueezeNet	89.77	89.31	89.69	89.50 ± 11.44	3.21	~1.3	~0.83	~4.4
VGG-16	91.25	90.76	89.85	90.31 ± 10.64	2.90	~138.0	~7.2	~12.4
DarkNet-19	93.53	93.98	94.06	93.97 ± 6.33	3.10	~20.8	~7.5	~20.5
Existing ViTs (Case 1: Performance on test original set)								
Tiny ViT	89.14	88.83	90.88	89.85 ± 11.10	5.65	~28.9	~7.3	~17.1
Cross ViT	92.97	90.28	95.59	92.86 ± 8.12	6.32	~44.0	~5.6	~16.2
LocalViT	93.08	92.83	94.59	93.70 ± 7.29	5.82	~27.5	~6.8	~17.5
Dual-ViT	94.52	95.73	93.7	94.88 ± 6.12	5.95	~12.4	~1.9	~7.2
(Hybrid ViT) LeViT	96.78	97.00	97.02	96.99 ± 5.26	5.20	~9.8	~4.5	~11.0

### 4.3 Comparison techniques

#### 4.3.1 Comparison with various setups

The NeuroCST architecture consists of parallel branches with optimized configurations. While maintaining the CNN and SwinLite-T component depths identical to NeuroCST, the classification performance was assessed using various stage configurations. Throughout framework training, hyperparameters play a crucial role. The weight decay in the Adam optimizer, the expansion ratio of the MLP in the ES-TB, and the number of feature mappings (channels) served as the specific hyperparameter settings utilized for assessment of NeuroCST classification performance.

#### 4.3.2 Comparison with existing methods

Examination of frameworks built upon prior robust 2D natural image classification core networks preceded evaluation of the proposed parallel dual-branch architecture. Computational overhead and parameter counts were additionally computed. All frameworks were compared under identical training approaches, data augmentation, and optimizers comparable to NeuroCST. Compared with CNN frameworks, transformer-based counterparts exhibit larger parameter counts and computational costs, as Table 4 demonstrates. Across the reported evaluation metrics, the proposed parallel dual-branch architecture achieves the highest results on the employed dataset, while its computational overhead is comparable to that of the 2D CNN frameworks.

The proposed NeuroCST framework demonstrated superior performance on the employed dataset across a wide range of measures. As presented in Table 4, NeuroCST achieved an accuracy of 98.18%, sensitivity of 97.92%, precision of 98.24%, and F1-score of 98.08% ± 3.11%. The enhanced spatial attention variant, NeuroCST-SA, achieved further improvements with an accuracy of 98.42%, sensitivity of 98.50%, precision of 98.60%, and F1-score of 98.55% ± 2.49%. The proposed NeuroCST framework outperformed existing CNN architectures, including InceptionResNetV2 (86.95%), GoogleNet (87.48%), InceptionV3 (88.59%), DarkNet-53 (88.13%), SqueezeNet (89.77%), VGG-16 (91.25%), and DarkNet-19 (93.53%). The framework also demonstrated superior performance in comparison with existing ViT architectures, including Tiny ViT (89.14%), Cross ViT (92.97%), LocalViT (93.08%), Dual-ViT (94.52%), and the hybrid LeViT (96.78%). Moreover, and precision–recall (PR) curves have been evaluated for binary dementia detection, with MildD, MD, and VMildD combined as the Demented class and ND as the Non-Demented class, as shown in Figure 4. The results demonstrate strong Demented-versus-Non-Demented discrimination, with NeuroCST-SA outperforming the evaluated CNN and ViT models. The confusion metrics have been drawn for the detailed comparison of the proposed NeuroCST-SA to assess robustness and generalization on various experimental setups (Figures 5–7). Case 1: evaluated on both the original test set and validation set, while in Case 2, all the data have been used from the original set. Regarding computational efficiency, NeuroCST-SA achieved an optimal balance between performance and resource utilization, with 14.8 million parameters, 4.9 GFLOPs, and an inference time of 11.4 milliseconds per image. No 3D model was trained in this study, so we make no quantitative comparison with 3D approaches; working on 2D slices is, however, expected to need less computation than volumetric processing. The ablation results in Table 4 indicate that the parallel fusion of the CNN and SwinLite Transformer branches, together with spatial attention, contributes to the improved performance of the framework.

Superiority in the performance of NeuroCST is due to the following critical aspects: (1) Parallel Dual Branch structure allows the capturing of localized features as well as global dependencies; (2) Feature Map Concatenation method combines complementary information without any increase in computational complexity; (3) Spatial Attention technique improves discrimination of intra-class differences; and (4) Lightweight architecture with separable self-attention and depth-wise separable convolution avoids overfitting.

## 5 Discussion

### 5.1 Experimental discussion

Numerous obstacles remain regarding widespread clinical deployment of such techniques despite encouraging findings from DL-based AD classification studies. The proposed NeuroCST architecture was developed upon a parallel dual-branch design integrating an enhanced EfficientNet-based CNN with a Lightweight SwinT. Three-dimensional algorithms were deliberately avoided, preventing a significant increase in the model’s parameter count and computational workload. The CNN branch captures localized morphological and textural patterns due to the convolutional receptive field. Moreover, NeuroCST employs several lightweight techniques including depth-wise separable convolution and separable self-attention facilitating deployment and training on low-performance machines. Whereas the SwinLite branch models broader contextual dependencies through hierarchical shifted window-based self-attention. Their feature maps are subsequently concatenated to preserve complementary local–contextual information. Overfitting mitigation and core network streamlining were achieved through the implementation of these lightweight techniques, in addition to data augmentation and the spatial attention mechanism (NeuroCST-SA variant). In NeuroCST-SA, post-fusion SA further refines the integrated representation by emphasizing discriminative spatial regions and suppressing less relevant or redundant responses using high weight for inter-class and balanced weight for intra-class variation. This architecture achieved an accuracy of 98.42% and an AUC of 0.986 on the AD-MID dataset following holdout-cross validation, substantially exceeding the performance of earlier studies. An ablation investigation served for assessment of lightweight approach effects upon model efficiency, parameter count, and computational burden. The lightweight techniques reduced the computational burden without lowering accuracy. The results in Table 4 show that accuracy rises step by step as the CNN branch, the SwinLite branch, and finally the spatial attention module are combined (97.31 and 97.74% for the single branches, 98.18% for NeuroCST, and 98.42% for NeuroCST-SA), while the parameter count (14.8 M) and FLOPs (4.9 G) of NeuroCST-SA remain in the same range as those of the standard SwinT baseline (12.4 M and 4.6 G).

As demonstrated in Table 4, omitting the CNN branch (SwinLite alone, which also lacks the spatial attention module) lowers accuracy by about 0.7 percentage points on both the Case 1 validation set (97.69% vs. 98.43%) and the Case 1 test set (97.74% vs. 98.42%). The similar size of the drop on both sets does not by itself show a generalization advantage. The inductive bias of the CNN branch is a plausible reason for the gain, but this was not tested separately. As such, the architecture is able to show commendable performance while being lightweight through the parallel integration of CNN and SwinLite Transformer branches. Independent testing set was used for the validation of the efficacy of the approach. The proposed method surpassed all the state-of-the-art frameworks in the comparison with a sensitivity of 98.50% and balanced accuracy of 98.50%, significantly surpassing the previous methods. Other metrics show that the proposed method outperforms or is comparable to the state-of-the-art frameworks. The results also show that the model’s feature encoding ability and generalization capability are improved through the parallel dual-branch architecture and spatial attention mechanism. Moreover, training of the lightweight NeuroCST architecture takes much less time (approximately 5.15 epochs per minute in the experiments; inference time 11.4 ms per image) and less computing power. Methods for risky region detection are based on 3D networks such as FCN and have been used in some recent research (Qiu et al., 2020) or for AD classification. By applying the identification methods, only high-variance regions are preserved to reduce unnecessary information in the following classification models. However, these approaches significantly increase the training cost. In turn, NeuroCST requires only one end-to-end training procedure.

### 5.2 Comparison with current ViTs and CNNs

Various CNN, ViT, and SwinLite architectures have been used for AD classification, including InceptionResNetV2, GoogleNet, InceptionV3, DarkNet-53, SqueezeNet, VGG-16, DarkNet-19, Tiny ViT, Cross ViT, LocalViT, Dual-ViT, LeViT (Hybrid), SwinT, and SwinLite. Accordingly, Table 4 displays the multi-class accuracy of the proposed model, with better performance compared to the traditional DL approaches on the same dataset. The proposed NeuroCST framework on the Case 1 original test set showed considerable enhancement in terms of accuracy by up to 4.89 and 11.47% when compared to traditional CNN models that were designed for local feature extraction, as shown in Figure 8. Specifically, NeuroCST-SA achieved an accuracy of 98.42%, substantially outperforming InceptionResNetV2 (86.95%), GoogleNet (87.48%), InceptionV3 (88.59%), DarkNet-53 (88.13%), SqueezeNet (89.77%), VGG-16 (91.25%), and DarkNet-19 (93.53%). Furthermore, the improvements in sensitivity were in the range of 4.52 to 18.78 percentage points, since NeuroCST-SA reached 98.50% compared to CNN sensitivity values of 79.72 to 93.98%. The improvements in precision were in the range of 4.54 to 14.02 percentage points, and the improvements in F1-score ranged from 4.58 to 16.41 percentage points, which indicates a better discrimination capability of the proposed model on this dataset.

**Figure 8:**
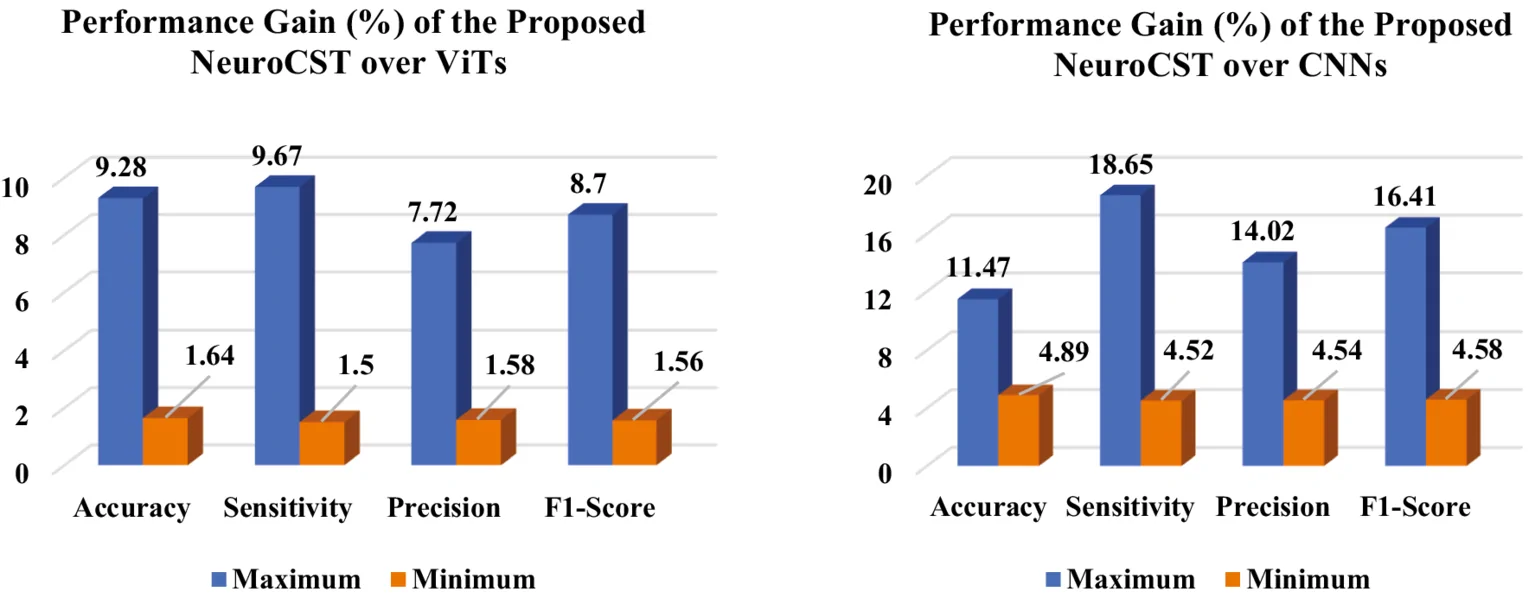
Performance improvements attained by the NeuroCST framework compared to traditional CNN, ViT, and SwinT frameworks (Case 1: performance on test original set).

Additionally, compared to existing ViT architectures, which focus on global feature extraction, NeuroCST-SA managed to achieve improvements in accuracy in the range of 1.44 to 9.28%. The proposed framework proved to be superior to Tiny ViT (89.14%), Cross ViT (92.97%), LocalViT (93.08%), Dual-ViT (94.52%), as well as hybrid LeViT (96.78%). Improvements in sensitivity over ViT models ranged from 1.50–9.67%, while improvements in precision ranged from 1.58–9.72% and F1-score improvements ranged from 1.56 to 8.70%. All these results prove that the parallel use of CNN and SwinLite Transformer branches, along with the spatial attention mechanism, provides a comprehensive combination of complementary local and global features, leading to superior diagnostic capabilities.

Moreover, it appears that SwinLite and SwinT models tend to outperform CNN and ViT models, showing high accuracy rates of 97.74 and 97.40%, respectively. The proposed SwinLite model showed comparable performance with 97.74% accuracy, 96.03% sensitivity, 98.00% precision, and an F1-score of 97.00% ± 4.02%, while having a lower number of parameters (9.6 M), GFLOPs (3.8), and a low inference time of 7.9 ms/image. While the hybrid LeViT model also managed to provide competitive results (accuracy rate 96.78%, sensitivity 97.00%, precision 97.02%, F1-score 96.99% ± 5.26%), NeuroCST-SA managed to get the best results in all the metrics, thus proving the superior fusion of enhanced local CNN-based feature extraction with global context modeling and spatial attention in the SwinLite Transformer branch. Finally, the lightweight nature of the proposed architecture makes it suitable for clinical settings with limited resources. NeuroCST-SA reaches the best performance-efficiency trade-off with 14.8 million parameters, 4.9 GFLOPs, and an inference time of 11.4 ms/image, providing superior diagnostic accuracy compared to conventional 3D models. A favorable accuracy-efficiency trade-off, along with superior multi-class classification of MildD, MD, ND, and VMildD patients, proves that NeuroCST is a promising base for a clinically deployable automated AD classification system.

### 5.3 Feature space visualization

In order to verify the effectiveness of the proposed framework, the feature maps of all the layers of the NeuroCST model were extracted based on the unseen test data. However, because of the high-dimensional features, visualization becomes difficult. Pooling layers and fully connected layers are followed by PCA for extracting features into 2D spaces. PCA is chosen because of its better ability to preserve the local geometry of the features owing to the nonlinear property of PCA, hence improving clustering and visualization quality. Figure 9 shows different segments of PCA, which demonstrate that the proposed NeuroCST-SA is able to extract more distinguishable features associated with MildD, MD, ND, and VMildD. The PCAs have been drawn to identify the discrimination across various experimental setups. Case 1: evaluated on both the original test set and validation set, while in Case 2, all the data have been used from the original set. In addition to this, this proves the effectiveness of the proposed parallel dual-branch CNN-SwinLite Transformer model along with the spatial attention mechanism for improved discriminative features. Higher separability between PC1 and PC2 of the proposed NeuroCST-SA suggests better feature discrimination.

**Figure 9:**
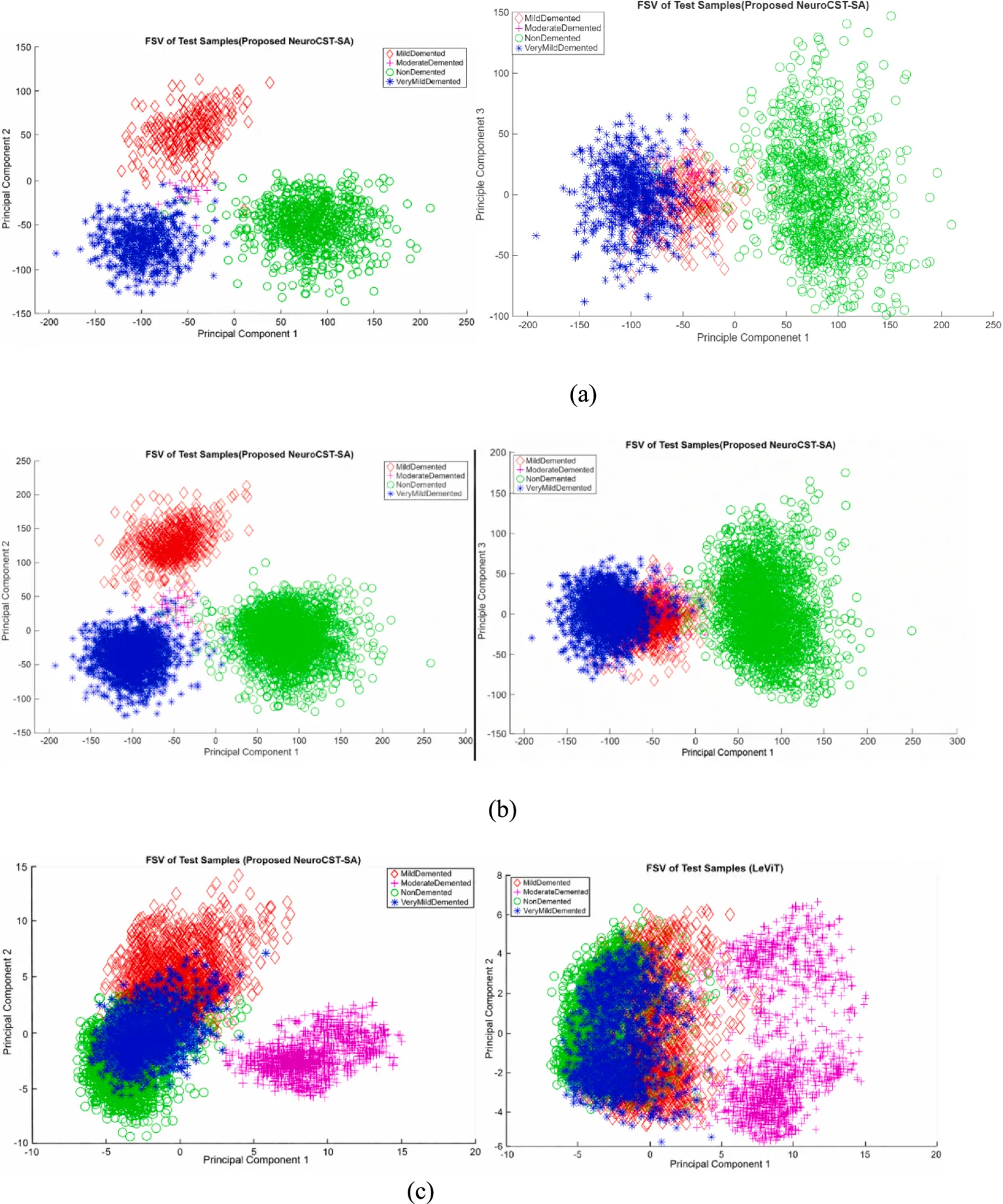
Illustrates some of the embeddings generated using the PCA technique in the following models: **(a)** the proposed NeuroCST-SA (Case 1: test original set); **(b)** NeuroCST-SA (Case 2: test original set); and **(c)** the proposed and current SwinLite (Case 1: validation set).

All the different AD classification categories are represented using different-colored points. From the findings of the PCA embeddings, it is clear that NeuroCST-SA provides highly distinguishable feature embeddings due to its excellent discrimination performance. The improved separation of the features is due to the collaboration between the parallel CNN branch used for local morphological feature extraction, the SwinLite Transformer branch, which is used for global context dependency modeling, and spatial attention for highlighting relevant diagnostic areas while filtering out redundant information. The separability of the features illustrated above is consistent with the quantitative results. It is evident from the embeddings that the NeuroCST framework captures the minute differences in morphology, contrast, and texture of AD across its four classification stages.

### 5.4 Limitations

This study has few limitations. First, NeuroCST works on individual 2D slices without inter-slice volumetric context; direct 3D processing also entails higher computational and memory costs, while many slices may contain limited disease-relevant information. Therefore, future work will explore ROI-guided 2.5D/3D analysis for more volumetric representation. Secondly, evaluation used Kaggle dataset derived from OASIS, without independent multicenter validation; thus, cross-site and patient-level generalization will be established. Finally, augmentation may introduce distributional dependence, while the absence of subject identifiers prevents verification of subject-independent splitting.

## 6 Conclusion

This study presents a parallel dual-branch architecture integrating an enhanced EfficientNet-based CNN with a Lightweight SwinLite Transformer, yielding NeuroCST, an efficient framework for automated AD classification from structural MRI. The parallel CNN branch provides inductive bias and captures fine-grained local morphological features characteristic of AD pathology, while the parallel SwinLite Transformer branch captures global contextual dependencies through its self-attention mechanism. Features from the two branches are combined by concatenation, giving rise to enhanced, complementary feature maps. A spatial attention mechanism is introduced in the NeuroCST-SA variant, focusing on important areas and neglecting irrelevant features, thus improving the discrimination between subtle differences between the classes. A few lightweight methods such as depth-wise separable convolutions and separable self-attention have been included, lowering the number of parameters and computations as compared to other state-of-the-art models which rely heavily upon computationally expensive 3D convolutions. The developed NeuroCST model gives results that are similar to or better than those of the state-of-the-art approaches; the NeuroCST-SA model achieves an accuracy of 98.42%, sensitivity of 98.50%, precision of 98.60%, and F1-Score of 98.55% ± 2.49%. It requires low computation with only 14.8 M parameters, 4.9 GFLOPs, and an inference time of 11.4 milliseconds per image. This work highlights the effectiveness of the developed approaches in achieving a compromise between computational overheads and diagnostic efficacy in automatic AD classification. Possible future research directions include the inclusion of non-image data like gender, age, and neuropsychological scores, along with multimodal data integration using imputation methods.

## Data Availability

Publicly available datasets were analyzed in this study. This data can be found here: https://www.kaggle.com/datasets/uraninjo/augmented-alzheimer-mri-dataset and https://data.mendeley.com/datasets/3r8hw8wmmk/1.

## Glossary

AD: Alzheimer's Disease
ADNI: Alzheimer's Disease Neuroimaging Initiative
AUC: Area Under the Curve
AIBL: Australian Imaging, Biomarkers and Lifestyle
BAC: Balanced Accuracy
CAD: Computer-Aided Diagnostic
CAT12: Computational Anatomy Toolbox
CNN: Convolutional Neural Network
DWC: Depth-wise Convolution
DSC: Depth-wise Separable Convolution
DTI: Diffusion Tensor Imaging
ECB: Efficient Convolution Block
ES-TB: Efficient SwinLite Transformer Block
FCN: Fully Convolutional Network
FME: Feature Map Enhancement
fMRI: Functional MRI
GELU: Gaussian Error Linear Unit
GAN: Generative Adversarial Network
GM: Grey Matter
MildD: Mild Demented
MD: Moderate Demented
MLP: Multilayer Perceptron
ND: Non-Demented
PET: Positron Emission Tomography
PCA: Principal Component Analysis
ROC: Receiver Operating Characteristic
ROI: Region of Interest
SEN: Sensitivity
SSA: Separable Self-Attention
SPC: Specificity
sMRI: Structural MRI
SwinLite: SwinLite Transformer
TL: Transfer Learning
VMildD: Very Mild Demented
ViT: Vision Transformer
WM: White Matter
W-MSA: Window-based Multi-headed Self-Attention
W-SSA: Window-based Separable Self-Attention
